# Injured worker experiences of insurance claim processes and return to work: a national, cross-sectional study

**DOI:** 10.1186/s12889-019-7251-x

**Published:** 2019-07-10

**Authors:** Alex Collie, Luke Sheehan, Tyler J. Lane, Shannon Gray, Genevieve Grant

**Affiliations:** 1 Insurance Work and Health Group, School of Public Health and Preventive Medicine, Monash University, 553 St Kilda Road, Melbourne, Victoria 3004 Australia; 2 Australian Centre for Justice Innovation, Faculty of Law, Monash University, Wellington Road, Clayton, Victoria 3770 Australia

**Keywords:** Return to work, Workers’ compensation, Insurance medicine, Claims management, Experience, Injury

## Abstract

**Background:**

Insurance claims management practices may have a significant impact on the health and experiences of injured workers claiming in workers’ compensation systems. There are few multi-jurisdictional studies of the way workers experience compensation processes, and limited data on the association between claims experience and return to work outcomes. This study sought to identify worker, claim and injury related factors associated with injured worker experiences of workers’ compensation claims management processes, and to examine associations between claims experience and return to work.

**Methods:**

A national, cross-sectional survey of injured workers involved in ten Australian workers’ compensation schemes. A total of 10,946 workers completed a telephone survey at 6 to 24 months post claim acceptance. Predictors of positive or negative/neutral claims experience were examined using logistic regression. Associations between claims experience, return to work status and duration of time loss were examined using logistic regression.

**Results:**

Nearly one-quarter (23.0%, *n* = 2515) of workers reported a negative or neutral claims experience. Injury type, jurisdiction of claim, and time to lodge claim were most strongly associated with claims experience. Having a positive claims experience was strongly associated with having returned to work after accounting for injury, worker, claim and employer factors.

**Conclusions:**

There is a strong positive association between worker experiences of the insurance claims process and self-reported return to work status. Revision and reform of workers’ compensation claims management practices to enhance worker experience and the fairness of procedures may contribute to improved return to work outcomes.

## Background

In most developed nations, compensation and rehabilitation of people with work-related injury or disease occurs primarily through social insurance or workers’ compensation systems. While approaches vary dramatically between and within nations [1, 2], these systems share common features, including applying eligibility criteria for claims and making payments for income support, healthcare and other benefits. These administrative functions are commonly exercised by a government authority or a private sector organisation acting as agent for government. These organisations typically interact directly with the injured worker and their employer and healthcare practitioners involved in the rehabilitation and return to work (RTW) process [2–4].

There is now substantial evidence that the policy and practices of the administering agency can have a significant impact on the health of injured workers. A systematic review of qualitative research on interactions between workers’ compensation insurers and injured workers reported that workers can experience these interactions as stressful and that this contributes to both poor mental health and loss of work function [5]. An Australian cohort study of people with traumatic injury reported that some administrative claims processes were identified as stressful by nearly one third of claimants, and that experiencing these processes as stressful was associated with elevated levels of disability and anxiety and depressive symptoms and lower reported quality of life six years after injury [6]. A systematic review of quantitative literature identified that aspects of the work injury claims administration process can impede return to work in some people [4]. These included delays in decision making, strict or rigid processes and poor communication. Conceptual models of work disability also suggest that administrative policy and procedure can impact RTW, through the settings and actions of insurance and compensation systems [7].

Given the wide coverage of insurance-based systems of injury compensation in society these effects may have significant impacts on public as well as individual worker health. In Australia alone there were nearly one quarter of a million new workers’ compensation claims in 2014 and more than 10.5 million workers were covered by the nation’s workers’ compensation systems [8]. In the same year, workers’ compensation systems in the United States provided coverage for an estimated 132.7 million workers and paid benefits totalling $62.3 billion [9].

Most studies on the relationship between insurance claim processes and injured worker health and RTW outcomes have been conducted in single jurisdictions or in small samples. There have been only a handful of quantitative studies [6, 10–13]. To our knowledge there have been no attempts to examine variations in claims experience between systems of compensation. There are also few studies that have specifically examined factors associated with injured workers’ claims experience [14, 15].

This study sought to examine both predictors of the injured workers’ experience of the workers’ compensation claims process, and the relationship between that experience and RTW. The study included a large sample recruited from ten different Australian workers’ compensation systems, each operating under independent legislative and regulatory models, and with a diversity of approaches to work injury claims management.

## Methods

### Setting and participants

Approximately 94% of Australia’s labour force of 12.5 million [16] are covered by one of the nation’s eleven workers’ compensation schemes [8]. Those exempt from requiring workers compensation insurance include workers who are self-employed, sole traders or independent contractors. These schemes provide benefits including wage replacement and healthcare for workers with injuries or other health conditions acquired in the course of employment [17]. The most common conditions covered by the systems include musculoskeletal disorders, minor trauma, mental health conditions and a range of occupational diseases including respiratory and circulatory system disease [17].

Each Australian state and territory has a single compulsory workers’ compensation scheme, and in addition there are commonwealth (national) schemes for federal government employees, some large national employers, maritime workers and defence force personnel. The primary objective of Australian workers’ compensation schemes is to return injured and ill workers to work. Despite their common objective, the Australian schemes also vary in many ways, including with respect to who in the labour force is covered by workers’ compensation insurance, what sorts of health conditions are eligible to receive support under the schemes, the duration and levels of income benefits provided, and in the methods of delivering those benefits [3]. Some relevant features of the schemes included in this study are described in Table 1, with much more detail on scheme differences available in an annual Australian Government report [17].

**Table 1:** Some features of the included Australian workers’ compensation schemes

Jurisdiction	Funding	Case Management	Maximum benefit duration	Responsible for RTW plan	% expenditure direct to worker	% benefit payments that are lump sum	Disputation rate
Queensland	Centrally funded	Single Public Insurer	104 weeks; 104–260 weeks (> 15% PI)	Insurer	64.4%	63%	3.3%
Tasmania	Privately Underwritten	Multiple private insurers	9 years (< 15% WPI); 12 years (15–20% WPI); 15 years (20–30% WPI)	Injury management co-ordinator	56.1%	60%	12.3%
Western Australia	Privately Underwritten	Multiple private insurers	Retirement age	Employer	54.9%	43%	3.8%
Seacare	Centrally funded	Regulator	Retirement age	Employer	66.6%	39%	31.3%
New South Wales	Centrally funded	Multiple private insurers	130 weeks (work capacity); 260 weeks (no work capacity)	Employer	47.2%	38%	4.6%
South Australia	Centrally funded	Multiple private insurers	104 weeks (most workers); Retirement Age (> 30% WPI)	Insurer	57.3%	57%	7.9%
Comcare	Centrally funded	Regulator	Retirement age	Employer	53.1%	9%	6.4%
Northern Territory	Privately Underwritten	Multiple private insurers	104 weeks (work capacity + suitable employment); 260 weeks (work capacity + no suitable employment); Retirement age (> 15% WPI)	Employer	62.8%	48%	8.1%
Victoria	Centrally funded	Multiple private insurers	130 weeks (most workers); Retirement Age (no work capacity)	Employer	52.9%	46%	11.6%

Note: Data shown are for the 2015/16 financial year; *WPI* Whole Person Impairment, *PI* Permanent Impairment

The schemes each fund or provide a range of services and supports to achieve this objective. Both employers of injured workers and the workers themselves have obligations to participate in return to work programs, although these obligations differ between jurisdictions. The claims handling or insurance functions of the schemes are performed either by private sector insurers or directly by government authorities. Healthcare and medical treatment is funded by the workers’ compensation schemes but is delivered via the national public health care system (Medicare) or through the private health care providers, and may include hospital, primary care, specialist medical, allied health and rehabilitation services.

The National Return to Work Survey (NRTWS) is commissioned by Safe Work Australia, a commonwealth government coordinating agency, on behalf of the Australian workers’ compensation authorities [18]. With the exception of the military and the Australian Capital Territory (ACT), all of the nation’s workers’ compensation authorities participate in the survey. The survey has been conducted annually since 1999 and biannually since 2014. This study includes data from the three most recent iterations of the survey conducted in 2013, 2014 and 2016. As per our prior analysis of the NRTWS data, eligible participants are injured workers with an accepted workers’ compensation claim who had taken at least one day off work and whose claim was submitted in the 24 months before the survey [19].

### Procedures

The NRTWS data is collected via computer assisted telephone interview, and data collection methods have been described previously [19]. Workers meeting eligibility criteria are identified from administrative claims data by workers’ compensation regulatory authorities. Contact details are provided to an independent survey company, which sends a letter describing the survey and providing options to opt-out of participating. Workers who do not opt-out are contacted via telephone and informed consent is sought. If consent is obtained the survey is administered immediately or at a later time nominated by the participant. The survey participation rate was 80% in 2013 and 2014, and 82% in 2016 [18]. This is calculated as the number of interviews ÷ (number of interviews + number of refusals). Information on when refusals occurred, and the number of people who opt-out prior to telephone contact was not available.

The NRTWS measures have been described elsewhere [18, 19]. They include self-reported indicators of return to work status (working at time of interview), workplace characteristics, employer and co-worker interactions, and experience of the compensation process and medical care, as well as worker occupational and demographic information. Survey data is linked to workers’ compensation claim data such as the date of condition onset and date of claim lodgement, and whether the worker is employed by a self-insured organisation or by an organisation insured through the jurisdictional compensation scheme.

### Outcomes

Two outcomes were defined for this study: (1) workers’ experience of the claims process and (2) self-reported RTW status.

Workers’ experience of the compensation claim process was assessed via a set of five questions, with responses measured on a five point Likert scale from 1 (strongly disagree) to 5 (strongly agree). A neutral response was indicated by a scale score of 3. The questions were consistent throughout all three of the survey periods as follows:

- “Thinking about the entire experience of being on workers compensation, I’d like you to tell me whether you agree or disagree with the following statements:
  1. The process was open and honest.
  2. There seemed to be good communication between the various people and organisations I dealt with.
  3. I felt like the system was working to protect my best interests.
  4. I believe the system treated me fairly.
  5. I feel that the system helped me with my recovery.”

Inspection of the data revealed a consistent response pattern with between 72 and 82% of the sample endorsing each of these statements (i.e., responding ‘agree’ or ‘strongly agree’), less than 3% of respondents providing neutral responses (i.e., ‘neither agree nor disagree’) and between 15 and 25% providing negative responses (i.e., ‘disagree’ or ‘strongly disagree’). More than 93% of workers who completed the survey responded to all five of the statements, and less than 2% of cases had more than 1 missing response.

To generate an overall rating of the claims experience for each worker in the sample, we calculated the average response for each worker across the five statements. The average was then categorised as positive overall response (mean score of > 3.0), or as a negative or neutral overall response (mean score of <=3.0). These cut points were chosen to best capture the bimodal distribution of the data, and to reflect the overarching response scale for each statement, where a score greater than 3.0 was rated as positive endorsement of the statement, a score of 3.0 was neutral and a score of less than 3.0 was negative.

Return to work status was recorded as whether the person was in paid employment on the day of telephone interview in response to the question “Are you currently working?” This was categorised into two outcome categories: (1) working at interview; (2) not working at interview [18].

### Predictors

Predictors were selected on the basis that they assessed demographic (sex, age), health status, injury type, occupational, employer, jurisdiction and claim factors. This was to ensure that a range of factors that had previously been reported as predictors of RTW and claims experience were included in analytical models [1, 20].

Self-rated health was collected on a 1 (poor) to 5 (excellent) scale and dichotomised into poor/fair/good and very good/excellent categories. Injury type was based on a modified version of the Type of Occurrence Classification System [21] to account for coding differences between the jurisdictions [22]. Injuries were classified as musculoskeletal (including both upper and lower body musculoskeletal conditions), fracture, mental health conditions, neurological (inclusive of brain and spinal cord injury), other trauma and other diseases (inclusive of respiratory, neoplasm, circulatory and skin diseases). Age was categorised into three bands: 15 to 35 years, 36 to 55 years, and 56 years or more. Jurisdiction of claim was recorded as the workers’ compensation scheme in which the claim was accepted. The time from date of acceptance of the workers’ compensation claim until the interview was categorised into 0 to 5 months, 6 to 11 months, 12 to 17 months and 18 to 23 months. Employer type was recorded as either premium payers (paying workers’ compensation premiums to the jurisdictional regulator) or self-insurer (funding own costs of workers’ compensation insurance). The time between injury onset and claim lodgement was calculated from administrative claims data and was categorised as less than 7 days, 7 to 13 days, 14 to 27 days, 28 to 55 days, 56 to 83 days, 84 to 180 days or more than 180 days.

### Data analysis

**Fig. 1:**
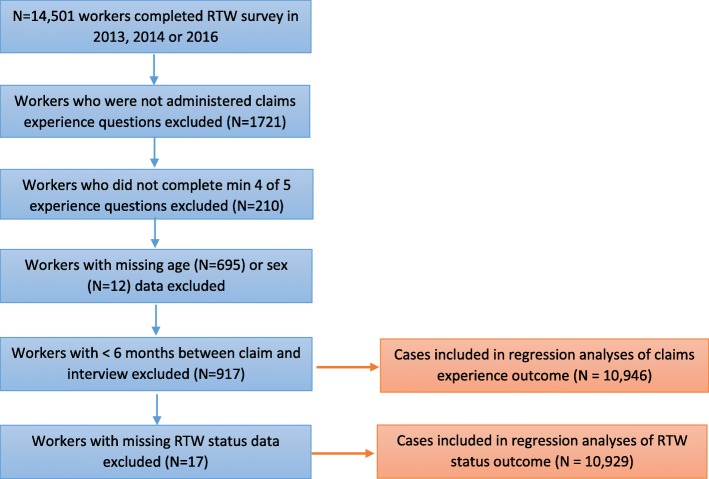
Overview of sample selection

Workers with missing gender and age were excluded, as were those who were interviewed but had not answered at least four of the five claims experience survey questions. Workers who lodged their claim less than six months prior to interview were also excluded from the analysis to enable a more accurate estimation of the time taken to RTW. Figure 1 provides an overview of the approach to cohort selection.

Frequency counts and percentages were used to describe the sample. Binary logistic regression was used to assess factors that were associated with worker claims experience. This model included the negative/neutral response as the reference category. Univariate analyses testing associations between each predictors and outcomes were conducted first. Predictors with *p* values less than 0.05 were included in the multivariate model, in which all predictor variables were included in a single step. All variables were retained in the final model. The adjusted odds ratio (AOR) represents the odds of an individual worker reporting a positive claims experience (relative to a negative/neutral experience) compared with the reference category for each predictor.

A second binary logistic regression model was used to assess factors associated with RTW status. In this model claims experience was included as an exposure variable in addition to the other predictors included in the first model. This model included the ‘not working’ status as the reference category. In this case the AOR represents the odds of a worker reporting that they were working at interview (relative to not working) compared with the reference category for each predictor. All analyses were carried out using SPSS v24.0 [23] with significance set at *p* ≤ 0.05.

## Results

### Overview of participants

A total of 10,946 workers were included in the final sample. Participant characteristics are summarised in Table 1. Just over half of the sample were aged between 36 and 55 years, with approximately one quarter in each of the younger and older age groups. Slightly less than two thirds of the sample were male, consistent with multiple other large Australian studies of workers compensation claims [1, 24]. Nearly two in five respondents rated their health as very good or excellent, with the remainder recording a rating of poor, fair or good. Nearly 60% of all cases involved musculoskeletal conditions, followed in prevalence by traumatic injury and fractures. Cases of mental health conditions comprised nearly 6% of the sample. Cases were dispersed across the workers’ compensation jurisdictions with larger number of workers from the larger jurisdictions of Queensland, New South Wales, Victoria, Western Australia and South Australia. The other, smaller jurisdictions have smaller samples with the smallest sample from the Seacare scheme with 258 respondents. Fifty five percent of claims were lodged within 14 days of injury onset, with nearly three-quarters being lodged within 28 days. Just over 90% of cases were from premium-paying employers. Nearly three-quarters of interviews were conducted between seven and eleven months post-claim onset, with smaller samples between thirteen and twenty-four months. Finally, the largest samples were from the two most recent iterations of the NRTWS in 2014 and 2016. Fewer cases were included from 2013, due to exclusion of cases with missing claims experience and age data, the majority of which were from the 2013 data file.

### Workers’ compensation claims experience

Seventy-seven percent of respondents reported a positive claims experience, with the remaining 23% reporting a neutral or negative experience. Binary logistic regression revealed that all predictors were statistically significantly associated with claims experience in the fully adjusted model (Table 2). Workers aged 56 years or older had 33% higher odds of reporting a positive claims experience than younger workers, after accounting for other factors. Male workers had 22% higher odds of reporting a positive claims experience than female workers, while workers with worse self-rated health had 63% lower odds of reporting a positive claims experience. There were multiple significant effects for both injury type and jurisdiction. Workers with fractures and other traumatic injuries had higher odds of reporting a positive claims experience than those with musculoskeletal conditions, in contrast to those with mental health conditions and other diseases which had significantly lower odds. Workers lodging their compensation claim with the Seacare, New South Wales, Northern Territory and Victorian schemes had significantly lower odds of reporting a positive claims experience than workers in the Queensland scheme. The time between injury/condition onset and claim lodgement had a significant effect, with delays of longer than 28 days associated with significantly lower odds of a positive claims experience. Workers employed by self-insured organisations, and those who were interviewed more than 18 months after claim lodgement, also had statistically lower odds of reporting a positive claims experience.

**Table 2:** Binary logistic regression testing factors associated with experience of the claims process

Factor	Sample Characteristics		Positive Experience		Negative or Neutral Experience		Binary Logistic Regression		
	N	col %	N	row %	N	row %	AOR	95% CIs	*p* value
Total	10946	100.0	8431	77.0	2515	23.0	n/a		
Age categor									
15 to 35 years	2849	26.0	2248	78.9	601	21.1	1.00		
36 to 55 years	5495	50.2	4110	74.8	1385	25.2	1.01	(0.90,1.14)	0.856
56 + years	2602	23.8	2073	79.7	529	20.3	1.33	(1.16,1.53)	< 0.001
Sex									
Female	3950	36.1	2897	73.3	1053	26.7	1.00		
Male	6996	63.9	5534	79.1	1462	20.9	1.22	(1.10,1.34)	< 0.001
General health									
Very Good/Excellent	4253	38.9	3705	87.1	548	12.9	1.00		
Poor/Fair/Good	6627	60.5	4678	70.6	1949	29.4	0.37	(0.33,0.41)	< 0.001
Missing	66	0.6							
Condition									
Musculoskeletal Conditions	6490	59.3	4927	75.9	1563	24.1	1.00		
Fractures	1346	12.3	1138	84.5	208	15.5	1.50	(1.28,1.77)	< 0.001
Neurological Injury & Disease	210	1.9	164	78.1	46	21.9	1.18	(0.84,1.67)	0.334
Mental Health Conditions	643	5.9	343	53.3	300	46.7	0.47	(0.39,0.55)	< 0.001
Other traumatic	1881	17.2	1559	82.9	322	17.1	1.30	(1.13,1.49)	< 0.001
Other diseases	365	3.4	293	80.3	72	19.7	1.13	(0.86,1.48)	0.389
Missing	11	0.1							
Jurisdiction									
Queensland	2183	19.9	1775	81.3	408	18.7	1.00		
Tasmania	1076	9.8	826	76.8	250	23.2	0.86	(0.71,1.04)	0.117
Western Australia	1491	13.6	1227	82.3	264	17.7	1.08	(0.90,1.30)	0.382
Seacare	258	2.4	193	74.8	65	25.2	0.65	(0.47,0.90)	0.010
New South Wales	1534	14.0	1180	76.9	354	23.1	0.77	(0.64,0.91)	0.002
South Australia	1329	12.1	1021	76.8	308	23.2	1.02	(0.85,1.22)	0.857
Comcare	785	7.2	558	71.1	227	28.9	0.85	(0.68,1.05)	0.126
Northern Territory	280	2.6	189	67.5	91	32.5	0.51	(0.38,0.68)	< 0.001
Victoria	2010	18.4	1462	72.7	548	27.3	0.74	(0.63,0.87)	< 0.001
Days to lodge claim									
Less than 7 days	3639	33.2	2962	81.4	677	18.6	1.00		
7 to 13 days	2382	21.8	1867	78.4	515	21.6	0.91	(0.79,1.04)	0.161
14 to 27 days	2151	19.7	1654	76.9	497	23.1	0.86	(0.75,1.00)	0.044
28 to 55 days	1379	12.6	1008	73.1	371	26.9	0.77	(0.66,0.91)	0.002
56 to 83 days	494	4.5	346	70.0	148	30.0	0.70	(0.56,0.87)	0.002
84 to 180 days	549	5.0	370	67.4	179	32.6	0.63	(0.50,0.78)	< 0.001
Over 180 days	351	3.2	223	63.5	128	36.5	0.60	(0.46,0.77)	< 0.001
Missing	1	0.0							
Employer type									
Premium-paying	9895	90.4	7689	77.7	2206	22.3	1.00		
Self-insured	1051	9.6	742	70.6	309	29.4	0.75	(0.63,0.88)	< 0.001
Time post claim									
6 to 11 months	8182	74.7	6365	77.8	1817	22.2	1.00		
12 to 17 months	1268	11.6	974	76.8	294	23.2	0.97	(0.83,1.13)	0.653
More than 18 months	1496	13.7	1092	73.0	404	27.0	0.78	(0.67,0.89)	< 0.001
Year of interview									
2013	2486	22.7	1885	75.8	601	24.2	1.00		
2014	4304	39.3	3304	76.8	1000	23.2	1.11	(0.97,1.26)	0.119
2016	4156	38.0	3242	78.0	914	22.0	1.17	(1.03,1.34)	0.018

Note: *AOR* Adjusted Odds Ratio, *CI* 5 and 95% confidence intervals, *p* value = level of statistical significance; *N* number, col.% = column percentage; row % = row percentage; Higher AOR correspond with a positive claims experience

### Return to work

Multiple predictors were associated with working status at the time of interview (Table 3). Of workers reporting a negative or neutral claims experience, 64.9% were working at interview, compared with 84.3% of those reporting a positive claims experience. Binary regression analysis showed that the adjusted odds of working at interview was 0.40 (95% CI: 0.35–0.44) for those with a negative or neutral claims experience compared to those reporting a positive experience. Most other predictors of RTW remained significant despite adjustment for claims experience in the same model, indicating that they are also independent predictors of the RTW outcome. For example, the effect of claims experience on RTW was approximately equivalent to that observed for workers with poorer self-rated health (AOR = 0.41; 95% CI: 0.36–0.46) when compared to workers with better self-rated health, and slightly larger than that for workers with mental health conditions (AOR = 0.49; 95% CI: 0.41–0.59) when compared to workers with musculoskeletal disorders. Older workers and female workers also had lower odds of working although the effects were smaller in magnitude. Compared to workers in the Queensland scheme, those claiming in New South Wales, Comcare and Victoria had significantly higher odds of working at interview, while those from the Seacare scheme had lower odds. A delay of greater than 84 days between injury onset and claim lodgement was statistically significantly associated with lower odds of working at interview, compared to claims that were lodged within seven days. Finally, workers employed by self-insured organisations had significantly higher odds of working at interview.

**Table 3:** Binary logistic regression testing factors associated with return to work status

Factor	Entire sample		Participants not working at interview		Participants working at interview		Binary Logistic Regression		
	N	col %	N	row %	N	row %	AOR	95% CIs	*p* value
Total	10929	100.0	881	8.1	1629	14.9	n/a		
Claims experience									
Positive	8419	77.0	1321	15.7	7098	84.3	1.00		
Negative/Neutral	2510	23.0	881	35.1	1629	64.9	0.40	(0.35,0.44)	< 0.001
Age category									
15 to 35 years	2848	26.1	520	18.3	2328	81.7	1.00		
36 to 55 years	5485	50.2	1068	19.5	4417	80.5	1.04	(0.92,1.18)	0.513
56 + years	2596	23.8	614	23.7	1982	76.3	0.75	(0.65,0.86)	< 0.001
Sex									
Male	6983	63.9	1477	21.2	5506	78.8	1.00		
Female	3946	36.1	725	18.4	3221	81.6	0.75	(0.67,0.84)	< 0.001
General health									
Very Good/Excellent	4249	38.9	455	10.7	3794	89.3	1.00		
Poor/Fair/Good	6614	60.5	1724	26.1	4890	73.9	0.41	(0.36,0.46)	< 0.001
Condition									
Musculoskeletal Conditions	6478	59.3	1304	20.1	5174	79.9	1.00		
Fractures	1346	12.3	253	18.8	1093	81.2	0.95	(0.81,1.11)	0.508
Neurological Injury & Disease	209	1.9	40	19.1	169	80.9	1.07	(0.74,1.54)	0.735
Mental Health Conditions	641	5.9	239	37.3	402	62.7	0.49	(0.41,0.59)	< 0.001
Other traumatic	1880	17.2	316	16.8	1564	83.2	1.09	(0.94,1.26)	0.251
Other diseases	347	3.2	42	12.1	305	87.9	1.85	(1.33,2.57)	< 0.001
Jurisdiction									
Queensland	2178	19.9	438	20.1	1740	79.9	1.00		
Tasmania	1075	9.8	210	19.5	865	80.5	1.21	(1.00,1.48)	0.056
Western Australia	1490	13.6	318	21.3	1172	78.7	0.97	(0.81,1.15)	0.711
Seacare	255	2.3	82	32.2	173	67.8	0.63	(0.46,0.86)	0.004
New South Wales	1533	14.0	273	17.8	1260	82.2	1.26	(1.05,1.51)	0.012
South Australia	1328	12.2	310	23.3	1018	76.7	0.92	(0.77,1.11)	0.392
Comcare	781	7.1	102	13.1	679	86.9	2.21	(1.70,2.87)	0.000
Northern Territory	280	2.6	61	21.8	219	78.2	1.14	(0.83,1.59)	0.417
Victoria	2009	18.4	408	20.3	1601	79.7	1.33	(1.12,1.57)	0.001
Days to lodge claim									
Less than 7 days	3635	33.3	673	18.5	2962	81.5	1.00		
7 to 13 days	2379	21.8	398	16.7	1981	83.3	1.16	(1.00,1.34)	0.055
14 to 27 days	2146	19.6	442	20.6	1704	79.4	0.86	(0.74,1.00)	0.053
28 to 55 days	1377	12.6	311	22.6	1066	77.4	0.81	(0.68,0.96)	0.017
56 to 83 days	493	4.5	117	23.7	376	76.3	0.81	(0.64,1.04)	0.100
84 to 180 days	548	5.0	139	25.4	409	74.6	0.72	(0.57,0.92)	0.007
Over 180 days	350	3.2	122	34.9	228	65.1	0.51	(0.39,0.67)	< 0.001
Missin									
Employer type									
Premium-paying	9880	90.4	2079	21.0	7801	79.0	1.00		
Self-insured	1049	9.6	123	11.7	926	88.3	2.16	(1.73,2.68)	< 0.001
Time post claim									
6 to 11 months	8168	74.7	1646	20.2	6522	79.8	1.00		
12 to 17 months	1265	11.6	226	17.9	1039	82.1	1.05	(0.88,1.24)	0.586
More than 18 months	1496	13.7	330	22.1	1166	77.9	0.91	(0.78,1.06)	0.241
Year of interview									
2013	2482	22.7	522	21.0	1960	79.0	1.00		
2014	4295	39.3	863	20.1	3432	79.9	0.96	(0.84,1.10)	0.557
2016	4152	38.0	817	19.7	3335	80.3	0.98	(0.85,1.13)	0.793

Note: *AOR* Adjusted Odds Ratio, *CI* 5 and 95% confidence intervals, *p* value = level of statistical significance, *N* number, col.% = column percentage; row % = row percentage; Higher AOR correspond with a returning to work

## Discussion

Insurance claims management practices can have a significant impact on the health of injured and ill workers involved in workers’ compensation systems [5, 6, 25]. This study presents new data from a large national study of injured workers’ experiences of the claims process and their RTW outcomes during the period 2013 to 2016. Self-reported claims experience was significantly associated with multiple personal, workplace and claim factors. The strongest associations were with health status, injury type and the duration of time between injury and claim lodgement. After adjusting for other factors, claims experience was significantly associated with self-reported work status. The magnitude of the association between claims experience and RTW was equivalent to or larger than that between RTW and other predictors entered in regression models.

This study supports and extends prior research on the intersection between worker claim experience, health and work function. Kilgour and colleagues’ [5] systematic review of qualitative studies of insurer-worker interactions identified that claim delays and claims management practices contributed to poorer mental health, social and vocational outcomes in some injured workers. Other studies have reported claims experiences as being associated with significant health concerns such as suicidal ideation [25], and have linked stressful claims experiences with poorer self-reported quality of life, disability and greater incidence of depressive and anxiety symptoms [6]. To our knowledge, this is the first study to examine claims experience across multiple workers’ compensation jurisdictions. Our findings demonstrate that the jurisdiction in which a worker lodged their claim was also statistically significantly associated with experience of the workers compensation process. Injured workers enrolled in the New South Wales, Victorian, Northern Territory and Seacare workers’ compensation schemes had statistically lower odds of reporting a positive claims experience than workers in the comparator jurisdiction of Queensland. Further examination of the claims processes in operation within and between jurisdictions is warranted, to identify specific contributors to claims experience.

These findings accord with our prior finding that the jurisdiction in which a worker makes a workers’ compensation claim is independently associated with duration of time loss [1], by extending this to self-reported working status. Workers enrolled in the Seacare workers’ compensation system were less likely to report being at work when interviewed than the comparator jurisdiction of Queensland, while workers in the New South Wales, Comcare and Victorian schemes were more likely to report being back at work. We previously proposed that these jurisdiction-level differences were likely due to variation in policy and practice [1]. The present study extends this finding to demonstrate that claims experience was independently associated with RTW outcomes, after accounting for jurisdiction of claim. This suggests that that they are at least in part independent constructs.

In insurance-based systems of injury compensation, such as the Australian workers’ compensation jurisdictions that were the subject of this study, government or private sector insurers are responsible for the administrative components of the workers’ compensation scheme. This includes responsibility for interacting with the injured worker and other participants in the RTW process [26]. Within this context, the perceived fairness of the administrative procedures has been identified as an important factor that can affect the health and work function of the injured person. For example, in a cross-sectional survey-based study of the experiences of long-term sickness absentees in the Swedish social insurance system, Lynoe and colleagues found that absentees perceived positive and respectful encounters with social insurance officers as facilitative of RTW, whereas negative encounters and the perception of being wronged impeded RTW [12]. Nordgren and Soderlund made similar findings in a survey of sickness absentees with heart failure [13]. In a study comparing cohorts enrolled in two different motor vehicle accident compensation schemes in Australia, claimant perceptions of the fairness of injury compensation processes were significantly associated with differences in self-reported health status [27]. Claimants in the system that was perceived as fairer also reported better physical and mental health at 12 to 24 months post-injury. The present study adds to the growing body of evidence that claimants’ perceptions of the fairness of procedures used in social insurance and compensation claims decision making can influence health and work outcomes. Features of just procedures include that they are unbiased, accurate, consistent, and that the affected person is involved or has ‘voice’ in decision making [28]. Review and reform of workers’ compensation claims processes to promote these features may improve workers’ claims experience and have a positive impact on RTW outcomes (23).

Our findings also provide further insight into the impact of claims management factors on claims experience and return to work outcomes. Delays of more than 28 days between injury or illness onset and the lodgement of the claim were associated with both a negative claims experience and lower odds of return to work. Prior studies have reported similar effects, albeit restricted to single jurisdictions. Cocker and colleagues [29] reported that delays in time taken to report, lodge and start wage replacement were associated with higher odds of prolonged periods of disability, in the Australian state of Victoria. Sinnott [30] found similar results in Canadian workers with low back pain. Our study demonstrates that this effect is also associated with experience of the claims process, and operates across multiple workers’ compensation jurisdictions. Reducing the time between injury onset and access to workers’ compensation system benefits may improve both claims experience and return to work.

Workers with mental health conditions were least likely to report positive claims experiences or be working at time of interview. These effects were observed after accounting for other factors in statistical models. This finding replicates prior studies of return to work in workers with mental health conditions [20], and may be a product of the assessment, diagnostic and management challenges that arise from the invisibility of the injury in these cases [31]. Multi-disciplinary workplace-based interventions such as work-focussed cognitive behavioural therapy have been identified as being effective at improving return to work outcomes in this group [32], and may assist to reduce the growing burden of mental health conditions in people of working age.

Self-reported health status showed strong associations with both claims experience and RTW. Those with poorer health were less likely to report positive claims experiences and less likely to have returned to work than those who rated their health as excellent or very good. Importantly, the association between health status and return to work was independent of claims experience, as both health status and experience were significant predictors in the RTW multivariate model, with approximately equivalent adjusted odds ratios. This finding reinforces prior research among injury compensation samples demonstrating relationships between health and claims experiences [6, 27] and a body of global evidence of the links between returning to work and health [33].

Paradoxically, we observed that workers employed by self-insured organisations had lower odds of reporting a positive claims experience but had higher odds of working at interview than workers employed by government-insured organisations. In most Australian workers’ compensation systems, employers can choose to underwrite and manage their own work injury claims if they meet certain thresholds related to company size and capacity to provide rehabilitation. In most jurisdictions between approximately 5 and 15% of the labour force are employed by self-insurers, with the exception of the Comcare scheme where the proportion of self-insured workers approaches 50% [17]. To our knowledge, this is the first published study to directly compare claim relevant outcomes between self and scheme insured organisations. Our findings suggest that self-insurers achieve superior work outcomes, however their workers are less satisfied with the experience of RTW. Closer investigation of the practices of self-insurers may help to identify aspects that can be adopted across the majority of the labour force employed by premium-paying employers.

The strengths of this study include the large sample, the coverage of multiple workers’ compensation jurisdictions, use of standardised outcome measures, and the inclusion of multiple claim, injury and psychosocial predictors. The cross-sectional nature of the data limits our ability to make causal inferences. Future research should examine changes in experience over time and the temporal relationship between claims experience and RTW outcomes. Other limitations include that the sampling strategy for the National RTW survey resulted in a sample biased towards longer duration claims, and that missing demographic data resulted in the exclusion of a large portion of the sample from analysis. Some findings contrast with our prior studies using administrative claims data. For example, we previously observed that injured workers from the state of Victoria had a longer duration of compensated time loss than workers in other states [1]; however, in the current study Victorian workers have higher odds of self-reported RTW than those in Queensland. Both the survey sampling strategy and the use of a different outcome measure may have contributed to this finding. The study was conducted in a nation with workers’ compensation arrangements that are similar to those in Canada and to some extent the United States, but quite different to those in other jurisdictions. This both limits the generalisability to settings with similar compensation arrangements, and provides impetus to explore insurance claims management experience in settings with different arrangements. The dataset did not enable us to estimate the relationship between claims experiences on the costs of workers’ compensation claims, which remains a knowledge gap. We were also unable to examine the impact of specific aspects of the claims process on either claims experience or RTW.

## Conclusions

In conclusion, this large study of injured workers with accepted workers’ compensation claims identified a strong and significant association between experience of the insurance claims process and RTW status. The findings suggest that jurisdiction of claim is independently associated with both claims experience and RTW, and that workers with mental health conditions and lower self-rated health status are more likely have both poorer claims experience and poorer RTW outcomes. Study findings have substantial implications for the delivery of insurance claims processes in workers’ compensation systems. Findings from prior studies suggest that approaches based on procedural fairness may be more likely to result in positive claims experience, and consequently to support positive return to work outcomes in injured and ill workers.

## Data Availability

The data that support the findings of this study are available from Safe Work Australia but restrictions apply to the availability of these data, which were used under license for the current study, and so are not publicly available.

## Abbreviations

AOR: Adjusted odds ratio
NRTWS: National return to work survey
RTW: Return to work
